# Medical Department of Our Army

**Published:** 1861-09

**Authors:** 


### EDITORIAL AND MISCELLANEOUS.

---

### Medical Department of our Army.

---

*To the Editor of the Chronicle & Sentinel:*

In your issue of the 13th inst., I notice an editorial on "the sufferings of our army," which must strike every reflecting man as just, opportune and to the point. The press of the land should speak out on this subject, and continue its discussion until the fatal neglect or incompetence which surrounds it shall be remedied. I beg your indulgence whilst I briefly call your attention to one of the departments upon which you animadvert, viz.: The medical department of our army:

If I am not incorrectly informed, this branch of the public service is radically wrong in its organization, or rather in its utter want of organization. In the first place, the Colonel of a regiment, who eftentimes secures his election by chicanery and electioneering, is the judge of the competency of his medical staff, and the selection is made not from the merits of the applicant, but for the amount of service rendered the Colonel in his election, or for other considerations not less objectionable. Consequently the physician of conscious worth, who cannot sacrifice his dignity and self-respect, and go into the squabble of an election, stands small chance of an appointment. Nor is the case improved in the instance of Independent Regiments where the appointment is made directly by the war department; for here also favoritism, and not ascertained merits, secures the commission. A regimental surgeon thus appointed is perfectly independent and free from supervision from any quarter; if he be incompetent or negligent there is no superior power to remedy the one or censure the other. Thus it is that there is neither order nor system, intelligence nor efficiency in the medical staff of the army: nor can such be expected until a thorough reorganization of the whole department shall be effected, and a central board of examiners established, before which each applicant shall undergo a strict and impartial examination. This plan, and nothing short of it, will secure for our brave volunteers competent medical men who understand the laws of health and their violation, as well as their manifestations of disease.

The establishment of hospitals at convenient points in Virginia may, and doubtless will remedy the evils adverted to. These at least can be efficiently organized and judiciously managed. But even in regard to these institutions, there is gross neglect somewhere. We have seen for weeks, in the Richmond press, statements that these hospitals are inadequately supplied with physicians, and that their inmates suffer in consequence: and that nurses are also needed. That state of things is unaccountable, and need not last 24 hours. Georgia alone would to-day furnish scores of competent physicians for positions in these hospitals, and the same may be said of the profession in the other States; the medical profession is ready and willing to discharge its duty to the country at any sacrifice. Why then, it may be asked, does this duty remain unperformed? Simply because professional services are not *officially* solicited, and when tendered, are not accepted. Many have sought these positions at the outlay of considerable expense and personal sacrifice in a journey to Virginia, and returned home in disgust, being unable to find even any one having authority in the premises, or possessing any information on the subject. Here, again, is the want of proper organization painfully manifested. Let those having authority in such matters duly announce the need of more physicians in Richmond, and in my humble opinion, the difficulty will consist in making selections from the great number of applicants. And as to nurses, there are medical students and young physicians enough in the Confederate States to furnish an abundant supply, on the simple announcement that they will be received.

PHYSICIAN.

We find the above timely comments on the medical department of the army in the Augusta *Chronicle & Sentinel* of the 18th September. The defects of this department have been of so glaring a character as to have arrested the attention of the most casual observer. Hence, we observe that the principal suggestion of this communication—the appointment of boards of examiners—has found its fulfillment in the following circular from the office of the *Surgeon General.*

---

Surgeon General's Office.

Richmond, Va., Sept. 27, 1861.

"Army Medical Boards, for the examination of Surgeons and Assistant Surgeons, have been ordered to convene at Norfolk, Richmond, Yorktown and Manassas.

Candidates for the appointment of Surgeons and Assistant Surgeons will be examined by these Boards, on presenting an invitation to appear before them from the Secretary of War, which may be obtained by forwarding their application, with testimonials of moral chrracter, to the War Department. Examining Boards will be held at other points farther South at a convenient time."

This is a move in the right direction, the more so that the regulation has a retrospective as well as prospective operation. As we understand it, every Surgeon and Assistant in the Medical service of the army is required to stand the test of an examination before a Board of competent Medical Examiners, &c.; if found incompetent, to be incontinently dismissed the service. These Boards, if they discharge their duties faithfully, will rid the army of the incompetent persons who have secured their positions upon considerations other than their true merits, and open vacancies for others who may be found qualified to fill them. Its prospective effect will prove salutary also in the highest degree. The medical aspirant for army honors will hereafter interrogate himself, not whether he can secure the appointment of the Colonel, but whether he is well posted in Anatomy, Physiology, Surgery and Practice. We are exceedingly gratified at this reformatory measure of the Medical Department of the Army, and are well satisfied that it cannot but result in greater efficiency in the service.

But what will become of the Regiments that are entering the State service? We would respectfully suggest to his Excellency, Gov. Brown, now that the State interests are entrusted to his keeping for another term, the appointment of a Board of Medical Examiners for the State, to judge of the competency of medical applicants for the State service.

